# Parental mental disorders and school performance among non-immigrant and second-generation immigrant children in Sweden

**DOI:** 10.1016/j.jmh.2025.100329

**Published:** 2025-03-27

**Authors:** Kenta Okuyama, Sara Larsson Lönn, Ardavan M. Khoshnood, Jan Sundquist, Kristina Sundquist

**Affiliations:** a Center for Primary Health Care Research, Department of Clinical Sciences Malmö, Lund University, Malmö, Sweden; b Department of Emergency Medicine, Skåne University Hospital Lund, Lund, Sweden; c University Clinic Primary Care Skåne, Region Skåne, Sweden

**Keywords:** School performance, Second-generation immigrant, Parental mental disorder

## Abstract

**Introduction:**

Immigrant children are often challenged at school. School performance is an important predictor of future socioeconomic position and mental and physical health. While studies have investigated parental mental disorders as a potential factor for poor school performance, no studies have investigated this among children with foreign-born parents, i.e., second-generation immigrant children. We aimed to examine whether parental depressive, anxiety, and personality disorders, affect school performance among non-immigrant children and second-generation immigrant children in Sweden.

**Methods:**

Multiple nationwide population register data in Sweden were used. Non-immigrant children, i.e., children born to two Swedish-born parents (*n* = 593,515), and second-generation immigrant children with two foreign-born parents from non-Western regions (*n* = 71,721) were included. School grades in the final compulsory school year were used as outcome. Parental mental disorders were measured in the inpatient and outpatient registers. While adjusting for potential confounders, the association between parental mental disorders and school grades was assessed by a linear mixed model. Interaction terms were included to examine whether the association between parental mental disorders and school grades differed by children's immigration status.

**Results:**

Parental mental disorder was associated with lower school grades for both non-immigrant and second-generation immigrant children and in both males and females. The school grades were lower among second-generation immigrant children but the effect of parental mental disorder was smaller among second-generation immigrant children than among non-immigrant children.

**Conclusion:**

Parental mental disorders affected the school performance of all children negatively. Future studies could examine what type of support at school for both second-generation immigrant children and non-immigrant children of parents with mental disorders are most beneficial.

## Introduction

According to the current estimate, 281 million people, i.e., 3.6 % of the world's population, are international migrants in 2020 (Mcauliffe and Triandafyllidou, 2021). This figure represents a significant increase compared to 173 million (2.8 % of the world's population) in 2000 and 84 million (2.3 % of the world's population) in 1970. As a result, many countries, which once used to be homogenous societies, have changed their demographic composition. Sweden is no exception; currently, 26.9 % of the national population has an immigrant background, i.e., foreign-born or born in Sweden with two foreign-born parents (Statistics Sweden 2023).

In general, children with foreign-born parents, even though the children themselves were born in the host country and have attained citizenship, have poorer school performance in comparison to non-immigrant children, possibly due to language and cultural barriers, and lack of assistance from their parents within school work (Finch et al., 2021). In Sweden, children who are born in the country but with foreign-born parents, i.e., second-generation immigrant children, have poorer school performance than non-immigrant children (Alvén, 2021; Taguma, 2010; Smith et al., 2019; Skolverket 2012; Skolverket 2023). School performance is important for children's future mental and physical health regardless of their immigration status. Poor school performance during childhood is associated with an increased risk of future mental disorders, e.g., psychosis, bipolar disorder and depression (Björkenstam et al., 2016; Gyllenberg et al., 2022; Isohanni et al., 1998; Jablonska et al., 2019), alcohol use disorders (Hayatbakhsh et al., 2011), and suicide (Björkenstam et al., 2011; Gunnell et al., 2011). School performance is also important for further educational attainment, employment, and higher socioeconomic position, which are strong predictors of subsequent health outcomes and well-being (Kawachi et al., 2010).

Several studies have investigated parental mental disorders as potentially important predictors of their children's school performance (Lin et al., 2017; Nordmo et al., 2023; Ranning et al., 2018; Shen et al., 2016). A nationwide longitudinal register study in Australia found that children of mothers with schizophrenia, bipolar disorder, unipolar depression, or psychosis had lower school grades at age 12 than children of parents without mental disorders (Lin et al., 2017). A register study in Denmark found that children of parents with schizophrenia or mothers with bipolar disorder had lower school grades at age 16 than children of parents without mental disorders (Ranning et al., 2018). A large register study in Sweden found that children of parents with depressive or mood disorders had lower school grades at age 16 (Shen et al., 2016). The most recent register study in Norway found that children of parents with depressive or anxiety disorders had lower school grades at age 16 even though the association was considerably attenuated after accounting for genetic confounding in sibling comparisons and restricted analysis on adoptees (Nordmo et al., 2023). Although it is yet inconclusive whether parental mental disorders are significant causes behind poor school performance due to unobservable confounders, e.g., genetic heritability of mental disorders and subsequent poor school performance, several studies have found potential pathways via environmental factors, e.g., impaired parent-child relationship and lack of parental assistance within school work (Ahun et al., 2022; Bechtiger et al., 2022; Pearson et al., 2016; Psychogiou et al., 2020; Ng-Knight et al., 2018).

Despite the elevated risk of poor school performance among children with foreign-born parents and children whose parents have mental disorders, few studies have investigated the association between parental mental disorders and school performance among children with immigrant backgrounds. Only two register studies in Sweden investigated parental post-traumatic stress disorder (PTSD) and school performance by mainly focusing on children with refugee backgrounds (Berg et al., 2022; Berg et al., 2019). The studies found that parental PTSD was associated with lower school grades at age 16 and higher ineligibility for secondary education among children who themselves or their parents were registered as refugees.

While these studies shed some light on the association between parental mental disorders and school performance among children with immigrant backgrounds, no large-scale studies have focused on the potential effect of more common parental mental disorders on children with foreign-born parents, i.e., second-generation immigrant children although the burden of more common mental disorders is increasing (Ten Have et al., 2023; GBD 2019 Mental Disorders Collaborators 2022; Winsper et al., 2020). In addition, depressive and anxiety disorders are some of the most common parental mental disorders to which children are exposed (Abel et al., 2019). If the relatively lower school performance of second-generation immigrant children is additionally affected by parental mental disorders, tailored interventions in these vulnerable groups may be needed.

The present study aimed to investigate whether parental mental disorders, i.e., depressive, anxiety, and personality disorders, differentially affect the school performance in second-generation immigrant children and non-immigrant children.

## Methods

This study was based on nationwide population and patient register data in Sweden, provided by Statistics Sweden and the National Board of Health and Welfare. To link data across different registers, pseudonymized serial numbers that replaced the unique 10-digit identification numbers that were assigned at birth or immigration to all Swedish residents were used. The replacement of the identification numbers with serial numbers was done by the authorities before we obtained the data. The following register data were used: the Register of the Total Population with yearly updates on sociodemographic data; the Multi-Generation Register (containing identification numbers of children born in Sweden or abroad after 1932 and identification numbers of their biological parents that can be used for linkages between children and parents); the Longitudinal Integration Database for Health Insurance and Labor Market Studies (LISA) with yearly information on education and household income 1990–2020; the Inpatient Register, containing hospitalization data in Sweden of Swedish inhabitants 1964 - 2020; the Outpatient Register, containing information from all outpatient clinics from 2001 – 2020 and the National School Register with grades from school year 9, the last year of compulsory school in Sweden. The study complied with the Strengthening the Reporting of Observational Studies in Epidemiology (STROBE) statement.

### Study subjects

Children who were born between 1997 and 2004 were included in the study. Based on parents' country of birth, children were defined as non-immigrant or second-generation immigrant children. Non-immigrant children were defined as those who were born in Sweden and where both of their parents were also born in Sweden. Second-generation immigrant children were defined as those who were born in Sweden and where both of their parents were born outside of Sweden, specifically non-Western regions, i.e., Africa; Asia and Oceania; Eastern Europe; Latin America and the Caribbean; and Middle East and North Africa. Children with one foreign-born parent and one Swedish-born parent were not included in the study.

### Outcome

School performance was obtained from the National School Register by the grade points at the end of compulsory school. In Sweden, the end of compulsory school is at the ninth school year when children generally turn 16 years old. The maximum grade points would be 320 if students achieved the highest grades, i.e., 20 points, for all 16 subjects.

### Primary exposure

Parental mental disorders were measured when children were between 7 and 15 years old, i.e., during the compulsory school years. We defined parental mental disorders from the Inpatient and Outpatient Register as maternal or paternal diagnoses using the following ICD-10 codes: depressive disorders (F32, F33), anxiety disorders (F40, F41, F43), and personality disorders (F60, F63, F68). Children of parents with other mental disorders were not included among the exposed or the unexposed non-immigrant and second-generation immigrant children.

### Potential confounders

Children's birth year was included in the analysis to account for age. The socioeconomic variables were measured when the children were six years old, i.e., before assessing parental mental disorders, to ensure temporality. Parental education was defined as low (compulsory school graduate), medium (high school graduate), or high (university graduate) based on the highest educational level among any of the parents. Data on parental education at age six were missing for a small number of individuals and to a slightly higher extent among second-generation immigrant children (Non-immigrant: 0.04 % (males), 0.03 % (females), Second-generation: 0.86 % (males), 0.79 % (females)). If parental education was available at a different age than 6 years and before 16 years of age, we used that data based on the assumption that the education level of parents is relatively stable over time. Otherwise, we categorized missing educational data into the low education category. Parental separation was defined using the family type variable and categories as living together or not (including both parental separation and death). Neighborhood socioeconomic status (SES) was defined as low, medium, or high based on the neighborhood deprivation index defined in Small Area Market Statistics (SAMS). SAMS is a commonly used neighborhood unit in Sweden and the detailed procedure of deriving the neighborhood deprivation index by SAMS can be found elsewhere (Winkleby et al., 2007). The data on neighborhood SES were missing slightly more among second-generation immigrant children (Non-immigrant: 1.33 % (males), 1.28 % (females), Second-generation: 5.64 % (males), 5.74 % (females)). We investigated whether the missing data would bias the results by conducting a series of analyses with or without the individuals with missing data. The results were similar between groups in terms of the direction and strength of the association between parental mental disorders and school performance (data not shown). However, since these individuals were very few, we excluded those with missing data on neighborhood SES from the analyses.

### Statistical analysis

First, we derived descriptive statistics of all variables by children's sex, immigration status, and parental mental disorders. To examine the association between parental mental disorders and school grades, we utilized linear mixed models and accounted for familial clustering by including a random effect for siblings with the same biological mother. First, the association between parental mental disorders and school grades was examined by adding the interaction term between parental mental disorders and immigration status. Second, the association between parental mental disorders and school grades was examined by adjusting for all included confounders and adding the interaction term between parental mental disorders and immigration status. In addition, the interaction terms between parental education and parental separation with immigration status were added to the model because the effects of each variable were largely different by immigration status, and they were affecting the association between parental mental disorders and school grades. In all models, the analysis was stratified by children's sex because the association between parental mental disorders and school performance has been indicated to be different between males and females according to previous studies (Shen et al., 2016; Ahun et al., 2022).

### Ethical consideration

This study was an observational study using pseudonymized nationwide, secondary data, released to us from the Swedish authorities. Ethical approval was obtained from the Swedish Ethical Review Authority (nr 2021-04268).

## Results

In total, 665,236 children were included in the study. Among them, 593,515 were non-immigrant children, and 71,721 were second-generation immigrant children.

Table 1 presents the descriptive statistics of all variables by children's sex, immigration status, and parental mental disorders. For both males and females, mean school grades were lower among children with parental mental disorders than children without parental mental disorders. In addition, among children with parental mental disorders, the percentage of high parental education levels was lower, and the percentage of parental separation and living in areas with low neighborhood SES were higher compared to children without parental mental disorders.

**Table 1:** Descriptive statistics of study subjects by children's sex, immigration status, and parental mental disorders.

	*Males*				*Females*			
	*Non-immigrant*		*Second-generation immigrant*		*Non-immigrant*		*Second-generation immigrant*	
	*Parental mental disorders*		*Parental mental disorders*		*Parental mental disorders*		*Parental mental disorders*	
	*No*	*Yes*	*No*	*Yes*	*No*	*Yes*	*No*	*Yes*
*Continuous variables, mean (S*D^1^)								
School grades	212.24 (57.1)	191.56 (67.86)	203.95 (61.99)	190.75 (68.94)	236.51 (57.46)	213.16 (70.7)	224.86 (60.79)	209.38 (68.8)
Birth year	1997.83 (2.65)	1998.06 (2.63)	1998.02 (2.65)	1998.1 (2.59)	1997.81 (2.65)	1998.03 (2.64)	1998.04 (2.65)	1998.12 (2.61)
*Categorical variables, n ( %)*								
Parental education								
High	139,468 (51.62 %)	1,4200 (41. 54 %)	1,1066 (38. 03 %)	2514 (34. 25 %)	132,902 (51.7 6 %)	1,3138 (40. 57 %)	1,0775 (38. 04 %)	2416 (34. 74 %)
Medium	126,160 (46.7 %)	1,8608 (54. 43 %)	1,3629 (46. 84 %)	3587 (48. 86 %)	119,570 (46.5 6 %)	1,7907 (55 .3 %)	1,3172 (46 .5 %)	3310 (47. 59 %)
Low	4536 (1.68 %)	1379 (4.0 3 %)	4401 (15.1 3 %)	1240 (16.8 9 %)	4312 (1.6 8 %)	1335 (4.1 2 %)	4382 (15.4 7 %)	1229 (17.6 7 %)
Parental separation								
Yes	37,945 (14.05 %)	9889 (28. 93 %)	5864 (20.1 5 %)	2098 (28.5 8 %)	35,859 (13.9 6 %)	9408 (29. 05 %)	5706 (20.1 4 %)	2012 (28.9 3 %)
No	232,219 (85.95 %)	2,4298 (71. 07 %)	2,3232 (79. 85 %)	5243 (71. 42 %)	220,925 (86.0 4 %)	2,2972 (70. 95 %)	2,2623 (79. 86 %)	4943 (71. 07 %)
Neighborhood SES^2^								
High	86,011 (31.84 %)	8549 (25. 01 %)	2059 (7.0 8 %)	443 (6.0 3 %)	81,114 (31.5 9 %)	7987 (24. 67 %)	2094 (7.3 9 %)	393 (5.65 %)
Medium	162,897 (60.3 %)	2,1182 (61. 96 %)	9325 (32. 05 %)	2304 (31.3 9 %)	155,282 (60.4 7 %)	2,0201 (62. 39 %)	8948 (31. 59 %)	2140 (30.7 7 %)
Low	21,256 (7.87 %)	4456 (13. 03 %)	17,712 (60.8 7 %)	4594 (62. 58 %)	20,388 (7.9 4 %)	4192 (12. 95 %)	17,287 (61.0 2 %)	4422 (63. 58 %)

1 SD: standard deviation. ^2^SES: socioeconomic status.

**Fig. 1:**
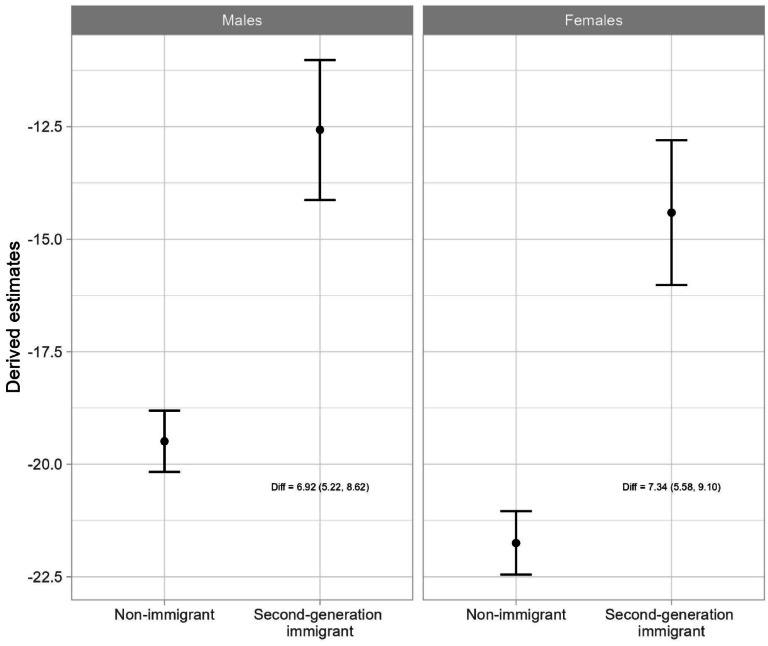
Association between parental mental disorders and school grades by sex and immigration status.

Table 2 presents the results of the linear mixed models examining the association between parental mental disorders and school grades including the interaction term of parental mental disorders and immigration status. The predicted mean value of school grades among children without parental mental disorders was 211.85 (males) and 236.00 (females) in non-immigrant, and 204.28 (males) and 225.04 (females) in second-generation immigrant children. For both males and females, parental mental disorders was associated with lower school grades. In addition, the negative effect of parental mental disorders significantly differed between non-immigrant and second-generation immigrant children. Specifically for males, being exposed to parental mental disorders was associated with −19.49 points lower school grades among non-immigrant children, and −12.57 points lower school grades among second-generation immigrant children. For females, being exposed to parental mental disorders was associated with −21.75 points lower school grades among non-immigrant children, and −14.41 points lower school grades among second-generation immigrant children. As a supplement to Table 2, Fig. 1 visually presents the estimated effects, i.e., the coefficient, of parental mental disorders on school grades for non-immigrant and second-generation immigrant children.

**Table 2:** Linear mixed model estimating the effect of parental mental disorders and its interactive effect with immigration status on school grades (Analysis was stratified by sex).

	*Males*	*Females*
	*Coefficient (95 % CI)*	*Coefficient (9 5 % CI)*
Intercept	211.85 (211.61, 212.09)	236.00 (235.76, 236.25)
Parental mental disorders (ref=No)		
Yes	−19.49 (−20.17, −18.81)*	−21.75 (−22.45, −21.04)*
Immigration status (ref=Non-immigrant)		
Second-generation immigrant	−7.57 (−8.33, −6.81)*	−10.96 (−11.74, −10.18)*
***Interaction with immigration status***		
Parental mental disorders (ref=No)		
Yes	6.92 (5.22, 8.62)*	7.34 (5.58, 9.10)*

⁎ Statistically significant results.

**Footnote:** Diff: Estimated difference in the association of parental mental disorder and its 95 % confidence interval. These are the estimated interaction terms in Table 2.

**Fig. 2:**
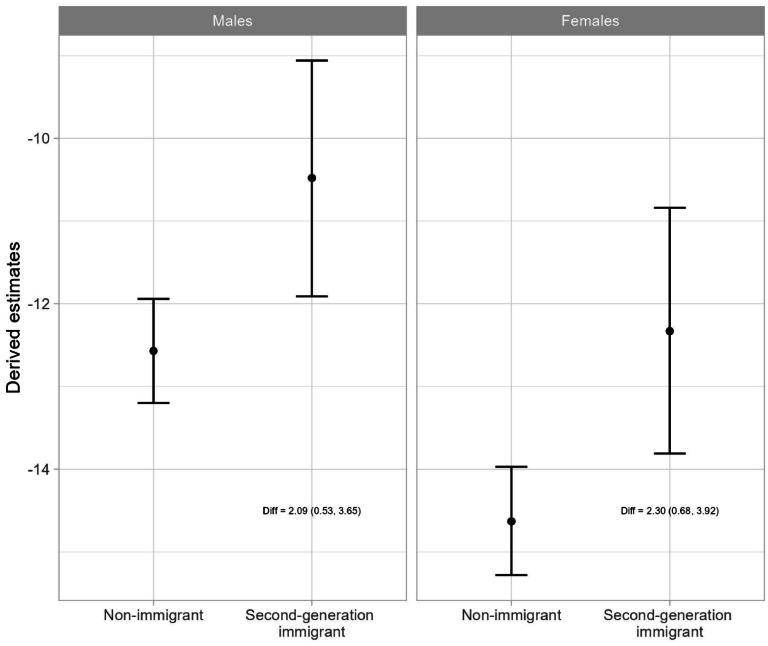
Association between parental mental disorders and school grades by sex and immigration status adjusting for potential confounders.

Table 3 presents the results of the linear mixed models examining the association between parental mental disorders and school grades after adjusting for all potential confounders and including the interaction terms between parental mental disorders, parental education, and parental separation and immigration status. Although the effect was attenuated after adjusting for potential confounders in both males and females, parental mental disorders were still significantly associated with lower school grades, and the negative effect differed significantly between non-immigrant and second-generation immigrant children by 2.09 points for males and 2.30 points for females. Specifically for males, being exposed to parental mental disorders was associated with −12.57 points lower school grades among non-immigrant children, and −10.48 points lower school grades among second-generation immigrant children. For females, being exposed to parental mental disorders was associated with −14.63 points lower school grades among non-immigrant children, and −12.33 points lower school grades among second-generation immigrant children. As a supplement to Table 3, Fig. 2 visually presents the estimated effects of parental mental disorders on school grades for non-immigrant and second-generation immigrant children. Although the effects of parental mental disorders were smaller among second-generation immigrant children, the predicted school grades were consistently lower among second-generation immigrant children compared to non-immigrant children.

**Table 3:** Linear mixed model estimating the effect of parental mental disorders and its interactive effect with immigration status on school grades adjusting for potential confounders (Analysis was stratified by sex).

	*Males*	*Females*
	*Coefficient (95 % CI)*	*Coefficient (9 5 % CI)*
***Main effects***		
Intercept	235 (234.53, 235.47)	256.88 (256.39, 257.37)
Parental mental disorders (ref=No)		
Yes	−12.57 (−13.2, −11.94)*	−14.63 (−15.28, −13.97)*
Immigration status (ref=Non-immigrant)		
Second-generation immigrant	−2.20 (−3.30, −1.10)*	−5.37 (−6.51, −4.24)*
Birth year (by year)	1.57 (1.51, 1.64)*	1.72 (1.65, 1.79)*
Parental education (ref=High)		
Low	−67.51 (−68.97, −66.04)*	−69.7 (−71.22, −68.19)*
Medium	−35.9 (−36.32, −35.49)*	−35.07 (−35.51, −34.64)*
Parental separation (ref=No)		
Yes	−16.3 (−16.84, −15.75)*	−16.42 (−16.99, −15.86)*
Neighborhood SES^1^ (ref=High)		
Low	−20.69 (−21.38, −20.00)*	−19.64 (−20.36, −18.93)*
Medium	−11.69 (−12.13, −11.25)*	−9.52 (−9.97, −9.06)*
***Interactions with immigration status***		
Parental mental disorders (ref=No)		
Yes	2.09 (0.53, 3.65)*	2.30 (0.68, 3.92)*
Parental education (ref=High)		
Low	24.07 (21.78, 26.36)*	28.62 (26.26, 30.97)*
Medium	15.78 (14.44, 17.11)*	14.63 (13.25, 16.01)*
Parental separation (ref=No)		
Yes	3.14 (1.67, 4.62)*	2.89 (1.36, 4.41)*

1 SES: socioeconomic status. *Statistically significant results.

**Footnote:** Diff: Estimated difference in the association of parental mental disorders and its 95 % confidence interval. These are the estimated interaction terms in Table 3.

## Discussion

The main findings of this study are that school performance in the final year of compulsory school is negatively affected by parental mental disorders in both non-immigrant and second-generation immigrant children. In addition, the effect of parental mental disorders on school performance was smaller among second-generation immigrant children compared to non-immigrant children. It is however important to keep in mind that the school performance of second-generation immigrant children was lower than that of non-immigrant children.

According to previous studies, there are two potential pathways between parental mental disorders and children's poor school performance that may apply to both non-immigrant and second-generation immigrant children. The first is an environmental pathway which could be attributed to the psychological burden of having parents with mental disorders, which in turn may impair parent-child relationship, school engagement, and exclusion from social contacts with peers (Ahun et al., 2022; Bechtiger et al., 2022; Pearson et al., 2016; Psychogiou et al., 2020; Ng-Knight et al., 2018). The second one is a genetic pathway which could be attributed to the genetic heritability of mental disorders and poor school performance (Nordmo et al., 2023; Psychogiou et al., 2020; Jundong et al., 2012). Although we did not investigate these pathways, our findings are consistent with previous studies (Lin et al., 2017; Nordmo et al., 2023; Ranning et al., 2018; Shen et al., 2016) suggesting that additional support for both second-generation immigrant children and non-immigrant school children is needed if parents have mental disorders. According to a systematic review and meta-analysis of randomized control trials of preventive interventions for children of parents with mental disorders, cognitive-behavioral therapy and psychoeducation at clinics or at home to help children and parents understand and cope with the parents’ mental health, and facilitate better communication were effective in the prevention of mental disorders among children (Lannes et al., 2021). Future studies could investigate whether certain interventions are uniformly effective for the school performance of second-generation immigrant and non-immigrant children.

One of our findings, i.e., that school performance of second-generation immigrant children was less affected by parental mental disorders compared to that of non-immigrant children, corroborates with the previous study which found that the effect of parental PTSD on school grades was smaller among children with refugee backgrounds compared to non-immigrant children (Berg et al., 2019). We could posit several reasons for this based on previous studies on school grades among second-generation immigrant children as well as the assimilation theory of second-generation immigrants.

First, second-generation immigrant children in general tend to perform worse at school than non-immigrant children due to several reasons rather than just parental mental disorders. Those include barriers in language, cultural understanding, and bullying ascribed to discrimination (Finch et al., 2021; Alvén, 2021; Taguma, 2010; Smith et al., 2019; Waters et al., 2010). These negative factors specific to second-generation immigrant children could have made the effect of parental mental disorders smaller than that of non-immigrant children. Our results showed that not only the effects of parental mental disorders but also parental education and parental separation on school grades were smaller among second-generation immigrant children than in non-immigrant children. This supports the hypothesis that other factors may exist affecting school grades specific to second-generation immigrant children. Nevertheless, this does not mean that parental mental disorders are unimportant for second-generation immigrant children's school grades. Their school performance was worse than non-immigrant children and was additionally affected by parental mental disorders.

Second, second-generation immigrant children might be more resilient to parental mental disorders compared to non-immigrant children. According to the segmented assimilation theory, second-generation immigrant children could succeed in upward social mobility in host societies by attaining education successfully despite their perceived disadvantages (Waters et al., 2010; Portes and Zhou, 1993). That could be achieved by parental aspiration for children's educational success or strong and positive social ties within neighborhoods with people from the same ethnic backgrounds. According to the Programme for International Student Assessment (PISA) report in 2015 and 2018 by the Organization for Economic Co-operation and Development (OECD), students with immigrant backgrounds reported a higher motivation to succeed in schools and beyond (OECD 2018; Cerna et al., 2021). Previous studies in Sweden found that immigrants had a reduced risk of mood disorders and drug use disorders by living in neighborhoods with a high density of migrants from the same origin (Dykxhoorn et al., 2020; Mezuk et al., 2019). These protective factors could have made the effect of parental mental disorders smaller among second-generation immigrant children than among non-immigrant children. However, the density of migrants from the same origin, so-called ethnic enclaves, in Sweden is relatively low compared to the highly segregated neighborhoods that were constructed over a long period of time in the US. In fact, the effects of ethnic enclaves on mental disorders and drug use disorders were quite small and limited to certain groups in Sweden (Mezuk et al., 2019; Mezuk et al., 2015).

Third, more integrated parents of second-generation immigrant children might have sought healthcare and got a diagnosis to a higher extent than less integrated parents. While universal and affordable healthcare is offered to all residents in Sweden, healthcare utilization might have been lower among parents who were less integrated into society. Potential differences in healthcare utilization have been indicated by register studies that found a lower prevalence or incidence of mental disorders among first-generation immigrants than among non-immigrant populations (Gadermann et al., 2022). As a result, the effect of parental mental disorders could have been reduced because children of more integrated parents would have performed better at school. However, we utilized inpatient and outpatient hospital records from specialist care, which capture more severe cases of mental disorders they were therefore assumed to be less biased by the integration level of the parents.

In summary, support for second-generation immigrant children of parents with mental disorders is needed because their school performance was generally lower than that in non-immigrant children and additionally affected by parental mental disorders. Future studies could investigate potential pathways by focusing on the ethnic composition in neighborhoods and how the integration level, e.g., length of stay, language and cultural proficiency of parents, may affect second-generation immigrant children.

### Strengths and limitations

One of the major strengths of this study was the use of population and patient data with high-quality information on socioeconomic and demographic characteristics, medical records of diagnoses, and school grades. Therefore, selection and information biases were low, and the findings should be generalizable to other countries with similar characteristics. Another strength was the specificity of the study population where we restricted children with immigrant backgrounds to second-generation immigrant children with two foreign-born parents from non-Western regions.

Despite these strengths, there were also several limitations. First, diagnoses of parental mental disorders might have been dependent on the healthcare-seeking behavior of individuals. There are possibilities that individuals, who are keeping up with public awareness of mental disorders, will obtain diagnoses to a higher extent. However, the data quality was validated as high concerning the prevalence of mental disorders by comparing with other data registers (Fazel et al., 2015; Sellgren et al., 2011), and our findings, i.e., a higher prevalence of mental disorders among foreign-born parents, are consistent with previous studies (Mindlis and Boffetta, 2017). We therefore believe that the degree of such bias is low. Second, our study did not account for types of schools, e.g., private and public schools, as well as different features of municipalities. In Sweden, all compulsory schools are subsidized by the government and required to follow the national curriculum. However, some private schools have specific focus on a certain subject, which may lead to differences between schools (Skolverket 2022). In addition, the compulsory school system is decentralized and administered by municipalities that may have different levels of diversity and local policies for migrants (Taguma, 2010). Not accounting for the type and features of schools and municipalities might have biased our results. Third, we did not have access to ethnicity in our nationwide data but rather country of birth. Therefore, our reference group, i.e., children born to two Swedish-born parents, could have included children with non-Swedish ethnicity.

## Conclusion

Parental mental disorders negatively affected the school performance of both second-generation immigrant children and non-immigrant children. In addition, second-generation immigrant children were less affected by parental mental disorders compared to non-immigrant children although their grades were lower. Support at school for children of parents with mental disorders would therefore be helpful for both second-generation immigrant and non-immigrant children but may need to be shaped differently.

## Funding

This project has received funding from the 10.13039/501100000781European Research Council (ERC) under the European Union's 10.13039/501100007601Horizon 2020 research and innovation programme (grant number 787592). This project has also received funding from The 10.13039/501100001862Swedish Research Council (grant number 2021-06467).

## CRediT authorship contribution statement

**Kenta Okuyama:** Writing – review & editing, Writing – original draft, Visualization, Methodology, Conceptualization. **Sara Larsson Lönn:** Writing – review & editing, Visualization, Supervision, Methodology, Formal analysis, Data curation. **Ardavan M. Khoshnood:** Writing – review & editing, Methodology. **Jan Sundquist:** Writing – review & editing, Resources, Funding acquisition, Conceptualization. **Kristina Sundquist:** Writing – review & editing, Supervision, Resources, Project administration, Methodology, Funding acquisition, Conceptualization.

## Declaration of competing interest

The authors declare that they have no known competing financial interests or personal relationships that could have appeared to influence the work reported in this paper.
